# Bibliographical Notices

**Published:** 1853-11

**Authors:** 


*A Practical Treatise on the Diseases of Children.* By D. FRANCIS CONDIE, M. D., Secretary of the College of Physicians, etc., etc. Fourth Edition, revised and augmented. Philadelphia, Blanchard and Lea, 1853. pp. 732.

Dr. Condie's excellent treatise on the Diseases of Children is so familiar to the American profession as a standard work, and it has on previous occasions been so fully noticed in our columns, that we need only now announce the recent appearance of a *fourth* edition. The author's great experience, extensive reading, and familiarity with foreign languages, peculiarly fit him for the execution of such a work; and its value is abundantly proved by the numerous editions which it has reached in a very short period. It is characterized by great clearness and lucidity of style and arrangement, sound pathological views, and precise and plain therapeutical directions. The author claims "to present only those remedies and plan of treatment which he has found from actual observation, made at the bedside of the patient, to be best adapted to relieve or remove the several forms of disease of which he treats. * * * He has not failed, however, o notice every remedy and plan of treatment which comes to us with the recommendation of practitioners of unquestionable authority, even although he may not have had an opportunity of testing its efficacy."

Dr. Condie's remarks on re-vaccination appear to us to present very sound views on this subject. He believes that the system, once fully infected with the vaccine disease, enjoys a "protection against the occurrence of small pox, which is unimpaired with the lapse of time." But he admits and advocates the necessity of re-vaccination, "as a test whether the susceptibility of the individuals to the variolous disease has been fully extinguished." Whatever theoretical view may be adopted on this subject, whether with Dr. Condie, we assume that in a certain number of persons there exists "a greater or less degree of susceptibility to variolous infection, [which is] left unextinguished, and which susceptibility augments in time," or whether we adopt the opinion, "that the protective powers of the vaccine infection become gradually impaired, as the individuals who have been subjected to its influence advance in age," we think the question of the practical value of re-vaccination is abundantly settled. Our own experience in re-vaccinations, in a large Institution of children, has resulted in about 11 per cent. of cases, in which the genuine vesicle was reproduced in individuals who were supposed to have been vaccinated, and had good scars. And our first experiments with re-vaccination in this Institution, led we think, to very striking evidence of its prophylactic virtues. Two cases of modified small pox occurred in the Institution, during the epidemic which prevailed in this city in 1851. Immediately on the appearance of the eruption, we re-vaccinated all the inmates (over a hundred and thirty.) In twelve, the re-vaccination was successful, a large number had sore arms, with more or less approximation to the genuine vesicle; but no other cases of varioloid or small pox occurred in the Institution.

---

*A Treatise on Diseases of the Heart.* By O'B. Bellingham, M. D. F. R. C. S. Member of the Court of Examiners of the Royal College of Surgeons in Ireland, etc., etc. Part 1. Dublin: Fannin and Co. pp. 252.

This work is a republication, with revisions and additions, of Clinical Lectures delivered by the author at St. Vincent's Hospital, Dublin. They appeared originally in the Dublin Medical Press, and London Medical Gazette; and, having attracted considerable attention, have been appropriately re-issued in a distinct form.

"The work consists of two Parts: the first contains a description of the healthy heart, its size, weight, and the measurement of its chambers and orifices; followed by sufficiently full details respecting its motions and sounds. The examination of the heart in disease is then entered upon, commencing with the physical signs; the general signs, and the secondary or remote symptoms of cardiac disease are described in succession. The second part is devoted to the individual diseases of the heart, which are arranged according as their seat is the investing membrane, the lining membrane, or the muscular tissue of the heart, followed by a description of the functional or inorganic affections of the heart."—*Preface, p.* 8.

Part second only has been issued. It will be found to present a clear, concise, and accurate account of the heart, with original and novel views of its motions and sounds in health and disease.

The author rejects both valvular action and muscular contraction as influencing the normal sounds of the heart, and accounts for both the first and second sound as the result of friction between the blood and the parietes of the orifices of the heart. We extract his explanation of the sounds at length.

" *First sound.*—The first sound of the heart we know is synchronous with the ventricular systole : in this act, the blood, compressed by the contraction of the powerful muscular walls of the ventricles, is propelled with considerable force into the aorta and pulmonary artery, the sigmoid and semilunar valves of which are suddenly elevated. In the rapid passage of the blood from a wider to a narrower area, there must be considerable friction between this fluid and the parietes of the arterial orifices ; quite sufficient, in my mind, to produce the prolonged first sound of the heart. This sound necessarily has a distinct character from the second sound of the heart, because the resistance to be overcome is so much greater, and the passage of the blood through these orifices is more gradual ; it is likewise more prolonged, because sound must be developed during the entire period that the blood is passing from the ventricles into the large arteries : and the slower the action of the heart, the more prolonged will this sound be.

*Second sound.*—The second sound of the heart we know is synchronous with the ventricular diastole : during this act the muscular fibres of the ventricles are relaxed, the cavity of the ventricles enlarges, and the walls of the ventricles re-expand ; the curtains of the auriculo-ventricular valves open, and there is a sudden influx of blood from the auricles through the auriculo-ventricular orifices. It is scarcely necessary to say, that it is not the contraction of the auricles which propels the blood into the ventricles at this period of the heart's action ; nor is the dilatation of the cavities of the ventricles the result of the entrance of the blood from the auricles, as some have supposed. It is not, either, necessary for the production of this sound that the diastole of the ventricles should be an active process like the systole ; the ventricles being hollow muscles, the state of relaxation of their muscular fibres is a state of dilatation of their cavities : hence a vacuum would be created in them if the auricles were not at this period full of blood ready to supply them, but, as the latter had been filling during the whole period of the ventricular systole, this cannot happen, and the blood passes through the auriculo-ventricular orifices in a full and rapid stream, and with sufficient force to generate sound.

That the blood enters the ventricles with considerable force, would appear from what has been observed in experiments upon animals, as well as in the human subject. Cruveilhier says that, in a case of ectopia of the heart in an infant, when the organ was grasped with the hand during the ventricular diastole, it was violently and forcibly opened,—so much so, that he was at first under the impression that the diastole was the active state of the ventricles.

The second sound of the heart is much shorter than the first sound because the relaxation of the muscular fibres of the ventricles in the diastole is rapid, and the motion is sudden and instantaneous. It has a different character from the first sound, because the blood has no impediment to overcome in entering the ventricles from the auricles."

That friction between the blood and parietes of the orifices of the heart must be an important element in producing the first sound of the heart, we have long believed, and, with the author, we have regarded it as an inconsistency "to reject this element as incapable of producing the normal sounds, while it is set down as the one which almost exclusively gives rise to the abnormal sounds of the heart. We cannot, however, subscribe to the opinion, that friction is the only element in causing the first sound of the heart, for we believe it to be produced by several elements. And we cannot at all understand, how the short, sharp second sound is to be referred to the same cause as the first. Dr. Bellingham dwells pointedly upon its "different character from the first sound," which he attempts to explain by the fact "that the blood has no impediment to overcome in entering the ventricles from the auricles."

We have been for some time disposed to lean to the opinion, that both the sounds and impulse of the heart are best explained by adopting the account of its actions which has been deduced from the observations of Dr. Robinson, of Virginia, and Mr. Beau, of France. According to the cases of these observers (new born infants in whom the breast-bone was wanting) the action of the heart begins with an *active* diastole or dilatation of the organ in all directions, during which its apex is projected upward and forward, and the *impulse* is produced. This active diastole is followed, without observable interval, by the contraction or systole. And the first sound is probably the result, 1, of the friction between the blood and the auriculo-ventricular orifices during the active diastole; 2, of the subsequent closure of the mitral and tricuspid valves; and 3, of the friction between the blood and the orifices of the aorta and pulmonary artery, during the systole; while, 4, the impulse, and also 5, the muscular contraction in the systole may contribute to this sound. The second sound, according to this view, is produced by the closure of the semilunar and sigmoid valves, after the systole.

These opinions have been urged with much ability by Dr. A. Stillé, in a work on General Pathology, and are now adopted by

Prof. Wood in his lectures at the University of Pennsylvania. They are certainly plausible ; and, although they require the confirmation of further observations, before they can be positively admitted, they appear to us to offer the best explanation of all the phenomena of the heart's action that has been presented.

Dr. Bellingham's chapters on the Examination of the Heart in Disease, and on the Signs of Cardiac Disease, present a faithful and graphic picture of what is known on these subjects, and on many points his views are striking and original. We shall look with anxiety for the speedy appearance of Part Second.

---

*Proceedings of the American Pharmaceutical Association, at the Annual Meeting held in Boston, August,* 1853. pp. 48.

We have read these proceedings with much interest, and we feel confident that the action of this Association is destined to accomplish great good. It has taken the highest ground on the subject of quack medicines, as will be seen both from the resolutions adopted, and negatived.

Those adopted were—

" Resolved,—That the American Pharmaceutical Association believe that the use and sale of secret or quack medicines is wrong in principle, and is in practice attended with injurious effects to both the profession and the public at large, and believe it to be the duty of every conscientious druggist to discourage their use.

" Resolved,—That this Association earnestly recommend to our pharmaceutical brethren to discourage by every honorable means the use of these nostrums ; to refrain from recommending them to their customers ; not to use any means of bringing them into public notice ; not to manufacture or to have manufactured any medicine the composition of which is not made public ; and to use every opportunity of exposing the evils attending their use, and the false means which are employed to induce their consumption."

The resolution lost carries a no less pregnant negative.

" The following resolution, [embodying an opinion of the Committee on the Inspection of Drugs,] was offered, and after much discussion decided negatively :

" Resolved, That we believe the Drug Law was never designed to exclude foreign secret or quack medicines through the Custom House, nor do we believe this justifiable however *desirable.*"

